# Acoustic and visual stimuli combined promote stronger responses to aerial predation in fish

**DOI:** 10.1093/beheco/arab043

**Published:** 2021-07-19

**Authors:** Juliane Lukas, Pawel Romanczuk, Haider Klenz, Pascal Klamser, Lenin Arias Rodriguez, Jens Krause, David Bierbach

**Affiliations:** 1 Department of Biology and Ecology of Fishes, Leibniz-Institute of Freshwater Ecology and Inland Fisheries, Müggelseedamm 310, 12587 Berlin, Germany; 2 Thaer-Institute, Faculty of Life Sciences, Humboldt-Universität zu Berlin, Philippstraße 13, 10115 Berlin; 3 Institute for Theoretical Biology, Department of Biology, Humboldt-Universität zu Berlin, Philippstraße 13, 10115 Berlin, Germany; 4 Bernstein Center for Computational Neuroscience Berlin, Humboldt-Universität zu Berlin, Philippstraße 13, 10115 Berlin, Germany; 5 Cluster of Excellence ‘Science of Intelligence’ (SCIoI), Technische Universität Berlin, Marchstr. 23, 10587 Berlin, Germany; 6 División Académica de Ciencias Biológicas, Universidad Juárez Autónoma de Tabasco, Av. Universidad s/n, 86150 Villahermosa, Tabasco, México

**Keywords:** bird predation, multisensory integration, predation risk, sensory cues, startle response

## Abstract

Bird predation poses a strong selection pressure on fish. Since birds must enter the water to catch fish, a combination of visual and mechano-acoustic cues (multimodal) characterize an immediate attack, while single cues (unimodal) may represent less dangerous disturbances. We investigated whether fish could use this information to distinguish between non-threatening and dangerous events and adjust their antipredator response to the perceived level of risk. To do so, we investigated the antipredator behavior of the sulphur molly (*Poecilia sulphuraria*), a small freshwater fish which is almost exclusively preyed on by piscivorous birds in its endemic sulfide spring habitat. In a field survey, we confirmed that these fish frequently have to distinguish between disturbances stemming from attacking birds (multimodal) and those which pose no (immediate) threat such as bird overflights (unimodal). In a laboratory experiment, we then exposed fish to artificial visual and/or acoustic stimuli presented separately or combined. Sensitivity was high regardless of stimulus type and number (more than 96% of fish initiated diving), but fish dove deeper, faster, and for longer when both stimuli were available simultaneously. Based on the system’s high rates of bird activity, we argue that such an unselective dive initiation with subsequent fine-tuning of diving parameters in accordance to cue modality represents an optimal strategy for these fish to save energy necessary to respond to future attacks. Ultimately, our study shows that fish anticipate the imminent risk posed by disturbances linked to bird predation through integrating information from both visual and acoustic cues.

## Teaser

When fish are attacked by birds from outside the water, visual and olfactory cues are often distorted or inaccessible. However, additional cues like sound of the attack impact become available. In our experiments, fish responded strongest with diving when visual cues from above-surface movement and an impact sound were perceived simultaneously. Each cue alone might not be sufficient to indicate a bird’s attack, but combined, fish may identify the disturbance as an immediate threat.

## INTRODUCTION

Predation is one of the strongest selection pressures in nature, as failure to avoid predators is often associated with death, injury, or fear-induced stress (Sih 1987; Preisser et al. 2005). This led to the evolution of a wide range of antipredator behaviors (Lima and Dill 1990; Krause and Ruxton 2002; Kelley and Magurran 2003; Caro 2005). A prerequisite for effectively avoiding predation is the prey’s ability to reliably detect predators and assess the risk they pose. Prey can rely on a variety of sensory inputs including visual, chemical, auditory, and tactile cues to decide whether and how to respond to disturbances. Due to different propagation properties, cues of such diverse modalities vary in how susceptible they are to environmental change and what information they can provide (Weissburg et al. 2014). While some cues merely indicate a predator’s presence, others can provide detailed information on the predator’s identity (Martin et al. 2010; Englund et al. 2012), proximity (Ferrari et al. 2006; Bhattacharyya et al. 2017), diet (Mathis and Vincent 2000; Smith and Belk 2001), orientation (Helfman 1989; Kent et al. 2019), and even its satiation level (Smith and Belk 2001). However, relying on only a single cue may be misleading as cues can be unreliable or unavailable in certain contexts. For example, some cues may be absent or masked even in the presence of a predator (e.g., turbidity affecting vision: Abrahams and Kattenfeld 1997; Leahy et al. 2011; sounds masked by noise: Zhou et al. 2019). Vice versa, cues indicative of a predator may be present also in its absence (e.g., movement of harmless biota, rain, wind, or waves: Dalesman and Inchley 2008; Caldwell et al. 2010; Carr and Lima 2010; Ben-Ari and Inbar 2014). In groups of prey, animals can also infer threat from social cues, but this introduces an additional challenge of having to distinguish predator-induced from unrelated conspecific actions (e.g., Lima 1995; Quinn and Cresswell 2005). By integrating multiple cues from one or several sensory modalities (i.e., multisensory integration; Partan and Marler 1999; Munoz and Blumstein 2012), prey can either confirm or further refine threat predictions, which enables them to grade the risk depending on available cues and direct antipredator effort toward the most dangerous disturbances (i.e., threat sensitivity, for example, in amphibia: Mathis and Vincent 2000; crustaceans: Dalesman and Inchley 2008; birds: Englund et al. 2012; insects: Ben-Ari and Inbar 2014; and fishes [see citations below]).

Threat detection can be particularly challenging across the terrestrial-aquatic interface as many cues are less reliable, incomplete or lost entirely from one medium to another (Chase 2000). For example, when aquatic prey is attacked by predators from outside the water, chemoreception that often allows prey to detect and identify water-borne predator odors cannot be utilized for air-borne scents. While visual cues usually provide the earliest warning in the sequence of aerial predation (Katzir and Camhi 1993), they are perceived with distortion, so predators approaching at low angles can stay undetected for longer (see Lotem et al. 1991 for the opposite refraction problem faced by herons). These visual limitations further hamper above-surface threat identification as many disturbances will overlap in characteristics (e.g., movements, shapes, colors, or sizes). Such ambiguity can often be resolved once other cues become available. For example, while larval newts were unable to visually distinguish predatory salamanders from harmless tadpoles, they directed their antipredator response exclusively toward the predator once chemical cues were available (Mathis and Vincent 2000). Similarly, aphids perceived visual or vibrational cues as unreliable predictors of threat and only initiated escapes when they occurred in combination with a more reliable threat cue (Ben-Ari and Inbar 2014). One unique aspect of aerial-to-aquatic predation is that predators have to enter the prey’s media, which will ultimately provide more reliable visual cues and also produce additional hydrodynamic (mechano-)acoustic cues (see Englund et al. 2012 for differences in aquatic/aerial/terrestrial predation of ducklings). Aquatic prey could associate this unique combination of visual and acoustic cues upon impact with high reliability of denoting an immediate threat and consequently increase their antipredator efforts.

Despite considerable empirical support for “threat sensitivity” in aquatic prey, most studies focused on predation at intra-ecosystem level (e.g., intra-aquatic, see damselfish–trumpetfish: Helfman 1989; mosquitofish–sunfish: Smith and Belk 2001; hermit crab–brown crab: Dalesman and Inchley 2008; roach–perch/roach–pike: Martin et al. 2010). Avian predation is widely recognized as a major source of fish mortality and stress (Kramer et al. 1983; Pitcher et al. 1988; Gallagher et al. 2016; Culumber 2020), and predator detection through the surface has been acknowledged as a major determinant of attack success (Katzir and Camhi 1993; Pratt and Franklin 2009). Still, most studies investigating risk assessment and antipredator behavior in bird–fish interactions examined predation in a binary fashion (i.e., presence/absence of a predator, replica or stimuli: see Pitcher et al. 1988; Katzir and Camhi 1993; Litvak 1993; Gallagher et al. 2016) rather than exploring different types or magnitudes of predator stimuli.

We address this gap by investigating the response of a small freshwater fish to visual and acoustic stimuli from simulated bird attacks. The sulphur molly (*Poecilia sulphuraria*) is a well-suited study organism to investigate the flexible adjustment of antipredator behavior to aerial predation. In their natural habitat, sulphidic springs in southern Mexico, these fish experience severe hypoxia and toxic hydrogen sulphide (H_2_S), for which they compensate with extensive use of aquatic surface respiration (Tobler et al. 2009; Lukas, Auer, et al. 2021). While at the surface, these fish are exposed to high risk of bird predation (Kramer et al. 1983; Riesch et al. 2010; Lukas, Auer, et al. 2021). In the absence of major aquatic predators (Tobler et al. 2008), the water surface becomes the riskiest place and so sulphur mollies form large shoals and react to disturbances with diving into the oxygen-free water column (Supplementary Video 1). However, excessive diving increases energetic expenditure and reduces time for oxygen uptake (Plath et al. 2007), and thus recognition of dangerous events through the surface should be paramount for these fish.

Here, we investigated the importance of visual and acoustic stimuli in aerial predator avoidance of the sulphur molly. Our first aim was to explore the diversity of disturbances to which sulphur mollies are exposed in their natural habitat. We expected threat identification to be hampered due to an abundance of attack-like but unrelated visual and acoustic cues (e.g., from non-piscivorous birds or falling fruit) diluting or masking important cues that otherwise identify an impending attack. In addition to anecdotal evidence, we quantified with which frequency fish experienced attacks (multimodal) and attack-unrelated overflights (unimodal visual). We then used this information to inform an experiment in which we tested whether fish increased their antipredator response with the number of predator stimuli by presenting each stimulus separately (unimodal) and combined (bimodal). More precisely, we presented small groups of fish with 1) a moving object above the surface, 2) a playback of an impact sound, or 3) both stimuli simultaneously. We evaluated whether predator stimuli triggered a diving response and/or modulated certain diving characteristics using a video-tracking approach. In line with the threat-sensitivity hypothesis, we expected the strongest response when stimuli were combined.

## MATERIALS AND METHODS

### Natural observations of bird cues

**Figure 1:**
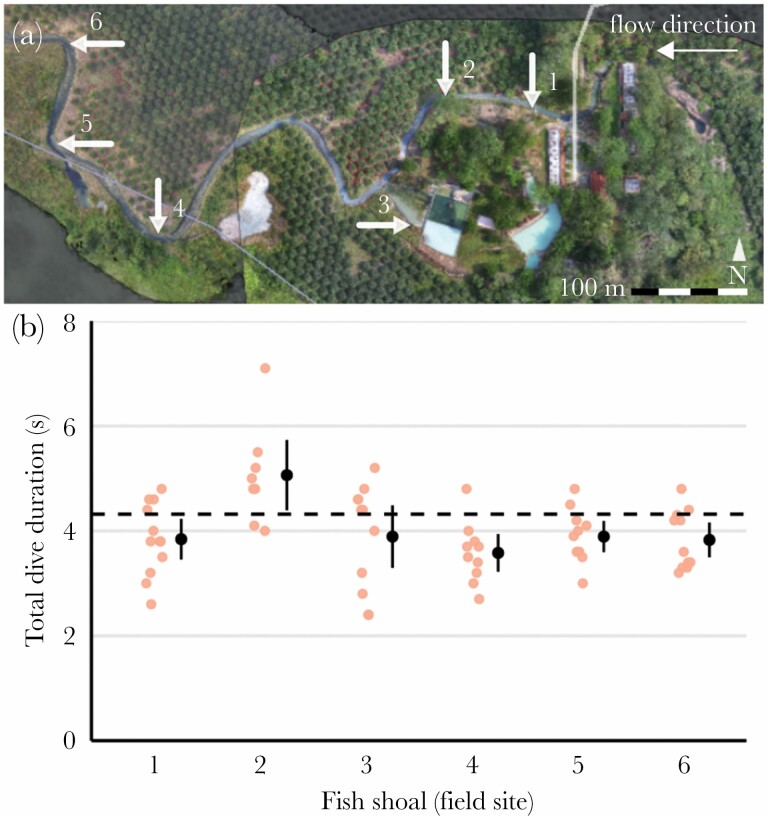
Sulphur mollies display a seconds-lasting dive response toward disturbance cues. (A) Aerial drone view of the sulphur spring system which *P. sulphuraria* inhabits. (B) Dive times in response to a visual predator stimulus observed at six sites along the sulphidic river habitat (LMM-estimated marginal means ± 95% CI). The dashed line indicates the global mean dive duration (4.32 s; LMM-estimated intercept).

We recorded disturbances that resulted in sulphur mollies diving (Supplementary Video 2; opposed to undisturbed diving of single individuals, see Lukas, Auer, et al. 2021) based on opportunistic daytime sightings at the Baños del Azufre spring complex (17°33′ N, 93°00′ W; April/May 2015–2019). In a subsequent survey, we evaluated how often fish would be exposed to bird attacks relative to an attack-unrelated activity such as bird overflights, which posed no immediate threat. We acknowledge that bird movements such as perch switches and walking ashore likely add a substantial amount of visual disturbances, but it was not feasible to reliably quantify these events. Using a similar approach previously described to quantify overall activity of bird predators in this system (Lukas, Auer, et al. 2021), we sampled a 50-m-long stretch of a 6-m-wide sulfidic stream of the Baños del Azufre, where sulphur mollies commonly aggregate (site 1 in Figure 1A). As predator activity varies throughout the day (Lukas, Auer, et al. 2021), we expected to find a similar pattern for the ratio of attacks versus overflights. For this purpose, 30-min surveys were performed during mornings (07:30–08:15), middays (12:00–13:30), and afternoons (15:45–17:30) on 4 consecutive days in May 2016. Attacks included all foraging attempts that resulted in a bird predator making water contact, and thus provided prey with both visual and mechano-acoustic cues (multimodal). Overflights included all incidents of predatory or nonpredatory birds flying through the predefined study area (below the tallest structure, < 3 m height) without making water contact, thus providing only visual cues (unimodal). We compared the frequency of both disturbance types using a generalized linear model with negative binomial error and tested whether disturbance activity varied throughout the day by including the two-way interaction disturbance type × sampling time (Supplementary Table 1; see Lukas et al. 2021 for reproducible R code).

### Design of artificial bird stimuli

Sulphur mollies are exposed to a diverse range of predators (see Results), which should favor a general recognition template for bird attacks. Thus, we chose to expose fish to general (opposed to species-specific) bird cues. Both the rapid expansion of an object (loom) as well as the use of computer-generated audio pips are established methods to reliably evoke antipredator responses in fishes (e.g., Bhattacharyya et al. 2017; McIntyre and Preuss 2019). To maintain some ecological relevance, stimuli were modeled on the hunting behavior of familiar bird species (see Supplementary Material for a description of hunting styles). For example, one of the major predators, the green kingfisher *Chloroceryle americana* is a small bird (body length: 217 ± 41 mm, single-wing length 93 ± 23 mm; Rodrigues et al. 2019) with white underbelly that attacks by plunge diving. Such a rapid predatory strike occurs over a relatively short temporal and spatial scale (opposed to prolonged chases from egrets), which is easier to replicate and manage in a laboratory setting. This inspired us to use a white styrofoam plate (210 mm × 300 mm as visual stimulus as well as an underwater recording of a projectile hitting the water as acoustic stimulus (duration: 1 s; see Supplementary Material for Audio file).

### Validation of artificial bird stimuli

An important prerequisite for our laboratory experiment (see below) was that wild sulphur mollies reliably responded toward the artificial bird stimuli with antipredator behavior. We thus exposed focal shoals to the visual predator stimulus at six locations of the Baños del Azufre spring complex (Figure 1A). The application of the acoustic stimulus was not feasible under field conditions, as the distance between shoals and the underwater speaker could not be standardized due to fish being disturbed by its placement and subsequently avoiding the speaker.

The visual stimulus was placed ashore (2.9–3.8 m distance to focal shoal) and operated remotely by releasing a mechanical spring mechanism. Upon release, the white plate loomed toward the fish for 1 s, before being returned to an undetectable position lying flat on the ground. We stimulated fish 10–12 times with a minimum of 60 s between exposures and recorded diving responses within a frame of interest that allowed for the detection of individual fish (Canon XF200 camcorder at 50 fps and full HD resolution). Fish sometimes swam out of frame as they increased the distance to the stimulus, which slightly decreased the sample size from 72 to *n =* 63 quantifiable stimulations. As the information of a detected threat often propagates through a group, we determined the interval from the point that 50% of fish had initiated diving until 50% had resurfaced. To quantify average dive times in response to a predator stimulus, we implemented a linear mixed model accounting for potential habituation from repeated stimulation (covariate “exposure”) as well as site-specific variation along the river (random effect “site”; see Lukas et al. 2021 for reproducible R code).

When exposed to an artificial visual predator stimulus in their river habitat, shoals of sulphur mollies responded with diving (Supplementary Video 1). Dives lasted on average 4.32 ± 0.26 s (model intercept ± SE; Figure 1B). Repeating stimulation up to 12 times did not affect dive duration (*exposure*: *F*_1,57_ = 3.6, *P* = 0.07; Supplementary Table 3). Stimulus-induced dives were considerably shorter than undisturbed (foraging) dives (~35–135 s; Lukas, Auer, et al. 2021) and fish showed a remarkably uniform response across shoals. We consider both facts as validation of our stimulus design.

### Diving response to predator stimuli in a laboratory setting

Experiments were conducted in a field laboratory in April 2017. Fish were collected each morning from the same site at which field observations had previously been carried out (site 1 in Figure 1A). Sample size was limited a priori to a total of 60 fish. Fish were visually matched for size (mean [range]: 15 [13–17] mm standard length; see Supplementary Material for size estimation procedure), resulting in similar body sizes across test groups (ANOVA: *F*_4,48_ = 2.1, *P* = 0.09). To recover from capture and handling stress, fish were kept in a cooler box containing water from the collection site and provided with aeration and filtration for at least 1 h.

**Figure 2:**
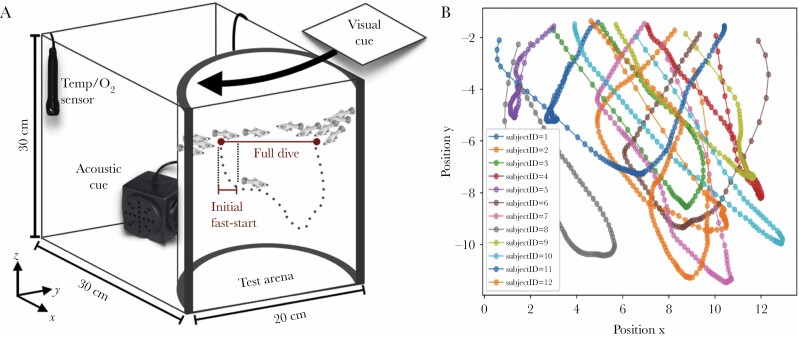
Experimental set-up. (A) Groups of fish were exposed to artificial predator stimuli (i.e., visual, acoustic, or bimodal) in a laboratory setting. (B) Individual diving trajectories were tracked to extract key diving variables.

On the basis of the sulphur molly’s shoaling behavior, we chose to investigate antipredator behavior in groups rather than testing single individuals (see evidence for group’s “calming effect”: Queiroz and Magurran 2005). Five groups of 12 individuals were haphazardly netted and introduced into the 12-L experimental tank. The tank was filled with water from the collection site to a water level of 25 ± 0.5 cm, which was renewed for each test group. Water conditions during the experiment were maintained at 28.5 ± 2°C and 0.6 ± 0.4 mg/L DO (monitored with OxyGuard Handy Polaris 2). To reduce outside disturbances and facilitate tracking, fish were confined to an opaque, semicircular arena (Figure 2A). Fish were acclimated to the arena for 10 min, a period that pilot tests deemed sufficient for the fish to resume their natural swimming behavior (i.e., fish swam close to the surface performing aquatic surface respiration; Lukas, Auer, et al. 2021).

We used three disturbance treatments that differed by stimulus number and/or modality (1 vs. 2 and visual vs. acoustic). For the *unimodal visual* treatment, we moved the white plate over the tank in a steady, looming motion. For the *unimodal acoustic* treatment, we used the playback of the projectile’s impact sound delivered by an underwater speaker (Daravoc® JH001 in the back compartment of the test tank). For the *bimodal* treatment, both stimuli were applied simultaneously. As fish during preliminary testing did not leave the surface without disturbance (i.e., control), we chose to compare effects between treatments. However, as it was not feasible to match the intensity of the visual and acoustic stimulus, this approach only allowed for a comparison between each unimodal with the bimodal treatment. We exposed fish to predator stimuli when they were situated at the surface. Test groups were stimulated 30 times. To minimize habituation to the stimuli, treatments were presented in randomized order and with a 60-s resting period between stimulations, which started once all fish had resurfaced. To avoid interobserver bias, the same team performed all stimulations in a systematic way.

Experiments were recorded using a Canon XF200 camcorder (full HD resolution at 50 fps) facing the front of the test tank. Diving trajectories in the XY-plane were tracked with EthoVision 12 (Noldus Information Technology, Wageningen, The Netherlands). Individual IDs were kept constant throughout a dive (i.e., tracking started 1–2 s before stimulus application until all fish had resurfaced), but could not be traced throughout a full experiment (i.e., between stimulations) as fish were visually occluded while swimming at the surface. The resulting trajectories were manually checked (0.006% could not be tracked reliably and were excluded) and processed using a custom Python script to perform a smoothing by moving average (i.e., window width of three frames) and to extract variables of interest. We acknowledge the loss of positional information in the Z-axis due to 2D tracking. While this constitutes a systematic error across treatments, we minimized this by 1) confining fish to an arena which promoted movement along the XY-axis instead (Figure 2A), and 2) choosing response parameters which were robust against this type of error (see below; with the exception of dive speed, which may be slightly underestimated).

A typical diving sequence consisted of an initial fast-start (see Domenici and Hale 2019), a small period of hovering at which the diving velocity decreased significantly and subsequent resurfacing, or, in some cases, a second dive (see Figure 2B for exemplary trajectory). To distinguish fast-starts from subsequent diving, we introduced a conservative threshold: fish were considered to have stopped their initial fast-start response when the velocity within two consecutive frames fell under 0.5 cm/s. Firstly, we assessed (1) *responsiveness* as the proportion of fish that responded to the treatment with diving. For all diving fish, we extracted additional diving characteristics: (2) *depth*, (3) *duration*, and (4) *maximum speed* of the initial fast-start. As a proxy for diving capacity, we further calculated (5) *total dive duration* from the moment a fish initiated diving until it resurfaced. Owing to the difficulties of keeping fish IDs across trials, dive characteristics (2–5) were reduced to group means.

We fitted (generalized) linear mixed models to analyze how the stimulus treatment affected responsiveness (binomial error), any of the three fast-start dive parameters (Gaussian error and REML estimation) and the total dive duration (log-normal error and REML estimation). We included *group ID* as a random factor to account for repeated testing of groups. We considered several candidate models, allowing for effects of the stimulus treatment as well as overall and stimulus-specific habituation. Based on AICc scores, the two-way additive model and the *stimulus*-only model received overwhelming support (Supplementary Table 4). However, as the *exposure* effect (i.e., trial number) was experimentally imposed, all estimates were based on the two-way additive model. Each model was validated by visual inspection of the residuals. Significance of fixed effects was based on likelihood ratio tests and pairwise comparisons of estimated marginal means were performed with mvt adjustment (using multivariate t-distribution). The full reproducible R code (R Core Team 2020) is available (see Lukas et al. 2021).

## RESULTS

### Natural observations of bird cues

We identified a total of 20 fish-eating bird species, including both obligate and facultative piscivores (Table 1; see Supplementary Material for a description of hunting styles). Predatory birds typically evoked a rapid, seconds-lasting dive response (Supplementary Video 2). Fish also occasionally dove in response to non-threatening species or events, such as the presence of non-piscivorous birds, reptiles, and insects or the movement of vegetation.

**Table 1:** Sources of disturbance which evoke dive responses in *P. sulphuraria*. Sources of disturbance have been identified primarily from opportunistic sightings during April–May, 2015–2019, and a systematic survey in May 2016 (total observation time: 360 min)

	Disturbance source	Total survey observation time (min)
*Fish-eating predators*	Wood-rails (*Aramides* spp.)	
	Sandpipers (*Calidris* spp.)	7.73
	Plovers (*Charadrius* spp.)	
	American pygmy kingfisher (*Chloroceryle aenea*) *	
	Amazon kingfisher (*Chloroceryle amazona*) *	7.17
	Green kingfisher (*Chloroceryle americana*) *	56.83
	Ringed kingfisher (*Megaceryle torquata*)	
	Great egret (*Ardea alba*) *	
	Cattle egret (*Bubulcus ibis*) *	
	Green heron (*Butorides virescens*) *	89.75
	Little blue heron (*Egretta caerulea*)	
	Snowy egret (*Egretta thula*) *	134.45
	Tricolored egret (*Egretta tricolor*) *	
	Black-necked stilt (*Himantopus mexicanus*) *	5.88
	Neotropical cormorant (*Phalacrocorax brasilianus*)	12.92
	Great kiskadee (*Pitangus sulphuratus*)	35.55
	Brown jay (*Psilorhinus morio*)	
	Great-tailed grackle (*Quiscalus mexicanus*)	295.13
	Bare-throated tiger heron (*Tigrisoma mexicanum*)	
	Shanks (*Tringa* spp.)	
*Predator-like disturbances*	Non-piscivorous birds (e.g., tyrant flycatchers, true thrushes, whistling ducks, ground doves, vultures)	
	Non-piscivorous insects (e.g., dragonflies, damselflies)	
	Non-piscivorous reptiles (e.g., basilisk, black iguana, green iguana, Morelet’s crocodile)	
	Wind/Moving vegetation	
	Water surface disturbance by uprising gas bubbles (H_2_S)	
	Human activity	

*Taxa previously reported by Riesch et al. (2010).

**Figure 3:**
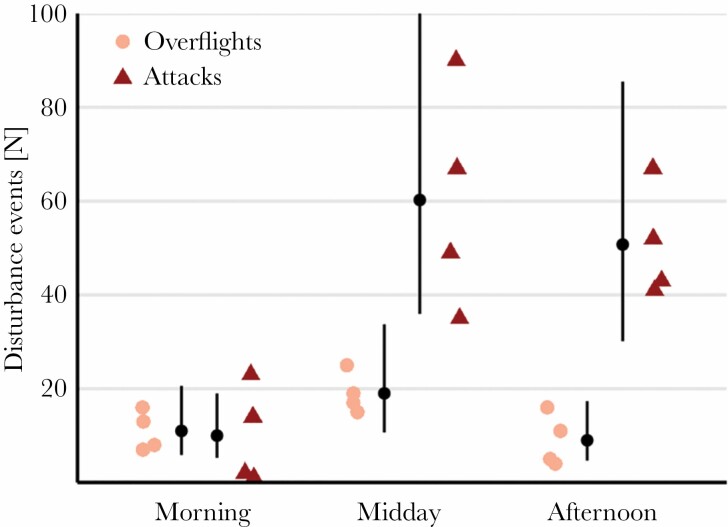
Sulphur mollies experience high rates of bird disturbances. Frequency of bird attacks and overflights (GLM-estimated marginal means ± 95% CI) observed during a 4-day survey of a 400-m^2^ stretch of a sulphidic stream (site 1 in Figure 1A).

Bird disturbances were frequent throughout the day (overall mean: 53 events per 30-min sampling; Figure 3). Generally, attacks were more frequent than overflights and fish experienced more disturbances later in the day (*disturbance type: F*_1,18_ = 25.6, *P* < 0.001; *sampling time: F*_2,18_ = 13.6, *P* < 0.001; for details, Supplementary Tables 1 and 2). However, the frequency with which attacks and overflights occurred changed throughout the day (interaction disturbance type × sampling time: *F*_2,18_ = 7.9, *P* < 0.001). During mornings, both disturbance types occurred at a similar rate (ratio: 0.9), while fish experienced more attacks than overflights during later parts of the day (ratio, midday = 3.2, afternoon = 5.6; Figure 3).

### Diving response to predator stimuli in a laboratory setting

**Figure 4:**
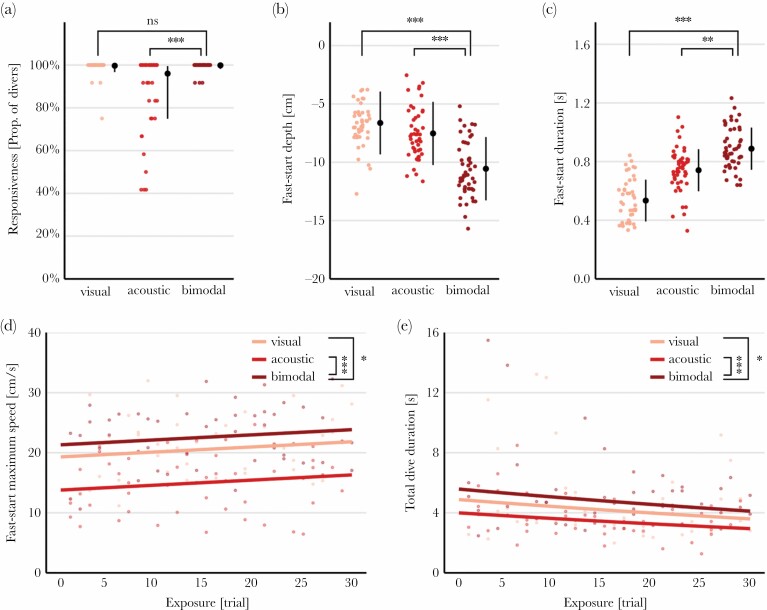
Exposure to different predator stimuli affected fish’s diving behavior. (A–E) Stimulus type affected fish’s responsiveness, depth, duration, and maximum speed of the fast-start response, as well as the total time fish spent underwater (*P* < 0.001). With repeated stimulus exposure, fast-start speed increased, while total dive time decreased (*P* < 0.03). Shown are group-pooled means (*n* = 5 groups of 12 fish) with model-estimated marginal means ± 95% CI or regression lines, respectively. Asterisks indicate results from post hoc pairwise comparisons (for details, see main text and Supplementary Tables 4–6).

Fish responded to all three predator stimulus treatments with fast-start diving. During unimodal treatments, fish initiated an escape in the majority of cases (responsiveness: visual 99.7%, acoustic 95.9%; Figure 4A), giving little margin for improvement when both stimuli were available (99.8%; but see difference acoustic–bimodal; effect of *stimulus*: *χ*^*2*^ = 91.2, *P* < 0.001). Fast-start characteristics differed significantly among stimuli treatments (effect of *stimulus*: depth *F*_2,135_ = 73.3, *P* < 0.001; duration *F*_2,135_ = 91.8, *P* < 0.001; maximum speed *F*_2,135_ = 51.3, *P* < 0.001). Presented with stimuli of both modalities simultaneously, fish showed deeper, faster and longer fast-start dives than after experiencing either stimulus alone (Figure 4B–D). Similar to the fast-start performance, fish remained under water for longer after bimodal relative to unimodal stimulation (effect of *stimulus*: *F*_2,135_ = 13.9, *P* < 0.001; Figure 4E). We detected no evidence for stimulus-specific habituation in any of the parameters (*stimulus × exposure* interaction, based on AICc scores; Supplementary Table 4), however, with repeated exposure to predator stimuli maximum speed increased slightly (covariate *exposure*: 0.08 ± 0.04 cm/s, *F*_1,135_ = 5.1, *P* = 0.026; Figure 4D), while total dive time decreased (covariate *exposure*: -0.01 ± 0.003 s, *F*_1,135_ = 10.8, *P* = 0.001; Figure 4E). We further observed that fish groups strongly synchronized during diving (Supplementary Video 1; see Supplementary Material for some explorative analyses of polarization and cohesion).

## DISCUSSION

In its natural habitat, the sulphur molly *P. sulphuraria* is exposed to disturbances of multifaceted nature and risk, but particularly fish-eating birds (up to 90 attacks in 30 min). A variety of bird and bird-like disturbances induced a rapid, seconds-lasting dive response in this species—a behavior that could be experimentally triggered in fish groups in a laboratory and field environment by imitating an above-surface visual cue (field and lab) and/or subsurface acoustic cue (lab). Our laboratory experiment revealed that fish initiated fast-start dives readily (high responsiveness) regardless of stimulus type and number, but modulated the magnitude of the dive response. For instance, fish performed deeper, faster and longer lasting dives when both stimuli were available simultaneously as compared to unimodal treatments. This finding is in line with previous work on threat-sensitive avoidance of aquatic predators (Helfman 1989; Smith and Belk 2001; Rieucau et al. 2014) and demonstrates that acoustic impact cues complement airborne visual cues in fish’s assessment of aerial risk. Ultimately, though, our results suggest that fish use this unique cue combination to anticipate avian risk levels and modulate the parts of their fast-start that are under behavioral control to optimize their antipredator response.

Sulphur mollies’ ubiquitous response toward a variety of bird(-like) disturbances appears to be diving (opposed to e.g., freezing). Such an avoidance of the surface both spatially and temporally is often considered the most effective way for aquatic prey to avoid avian predators (Kramer et al. 1983; Kersten et al. 1991; Rodríguez-Prieto et al. 2006). Our results show that the decision to dive (Figure 4A) is sensitive to both visual and acoustic predator cues. Regardless of whether a single or multiple cues of one or more modalities are present (Bhattacharyya et al. 2017; McIntyre and Preuss 2019), cue inputs in fish converge at the level of Mauthner neurons (or segmental homologs). Above a threshold, stimulation results in a single action potential that is translated into motor action leading to a so-called fast-start response (Domenici and Hale 2019 and references therein), which is essentially an all-or-nothing response. This suggests that all cues used in our study (both in the lab and field) exceeded the threshold level for dive initiation. The acoustic stimulus evoked significantly fewer dives than when the visual stimulus was available (unimodal and bimodal), but this effect was only marginal and likely resulted from differences in stimulus intensity. During a natural attack sequence, however, visual cues often become available prior to an attack (and therefor prior to acoustic impact cues) and imply a certain proximity. Such temporal and spatial encoding of cues will be most relevant for the initiation of evasive actions, which is an aspect of our research that will require further study.

Birds were found to attack at a rate of 1–2 attacks/min (this study; also Riesch et al. 2010; Lukas, Auer, et al. 2021), but harmless overflights represented a substantial proportion of the detected disturbances in this system (~17–52%, Figure 3). On the basis of visual cues alone, approaching predators are hard to distinguish from overflying birds without attack intention or non-piscivorous ones (Supplementary Video 2). Likewise, acoustic impact cues may alert fish to an actively hunting predator, yet can also result from falling fruit or branches. Bird attacks typically comprised visual and acoustic components, so, by contrast, the occurrence of both cues simultaneously could greatly reduce this ambiguity. Nonetheless, we found that response rates to all stimulus treatments were at (near)maximum level (96–99% of fish initiated diving). Moreover, wild fish frequently dove in response to moving vegetation, shadows or even ovipositing dragonflies, thus interrupting aquatic surface respiration even in the absence of a bird attack (false positive reaction). High sensitivity, as observed here, is often associated with the cost of increased false positive reactions (see Wolf et al. 2013), yet fish that fail to respond to a predator attack (false negative reaction) would be preyed on in nature. In groups of prey, individuals usually benefit from enhanced predator detection, numerical and/or behavioral dilution of predation risk (Krause and Ruxton 2002) and greater decision-making efficiency (Ward et al. 2011), all of which reduce the likelihood of predation for the individual and thus should result in a different optimal strategy compared to solitary prey. While the high predation pressure in this system may represent a strong selection regime acting on the predator sensitivity of sulphur mollies, it remains unclear whether the related responsiveness is a characteristic of this species and its extreme environment or a factor enhanced by their highly social lifestyle (which we simulated in our experiments through the use of groups).

Our laboratory experiment showed that dive depth, duration and speed differed among stimulus types with stronger diving responses following multiple stimuli. We interpret this as the ability of sulphur mollies to differentiate between dangerous and non-dangerous contexts on the basis of number of available cues. Similar observations have been made for aquatic predation of fish. Mosquitofish (*Gambusia affinis*) used visual and olfactory cues to increase their distance to the most dangerous fish predators (i.e., hungry and fish-fed; Smith and Belk 2001). Likewise, large schools of Atlantic herring (*Clupea harengus*) increased vertical avoidance when exposed to vision and motion of a submerged predator model than after unimodal models (Rieucau et al. 2014). Only recently it was shown that components of the fast-start response are under behavioral control and can be modulated by the perceived risk of aquatic predation cues (i.e., visual and odour; Ramasamy et al. 2015). Our results now add to this notion, demonstrating that fish also modulate their fast-start response under an avian predation scenario by integrating visual and acoustic cues.

The adaptive significance of such enhanced dives against aerial predators is exemplified by observations from an aviary, which revealed successful escapes to be generally deeper and faster (Katzir and Camhi 1993). Sulphur mollies’ stronger dive responses during bimodal stimulations likely increased fish’s escape probability when at risk. However, in a next step, different dive responses need to be linked to fitness benefits. Diving under the highest threat intensity (bimodal) ceased at a depth of about 11 cm, and lasted only seconds (<6 s) both in the lab and in the wild. While studies on the strike depth of kingfishers (Katzir and Camhi 1993) and herons (Lotem et al. 1991) suggest that the observed relatively shallow dives may not be deep enough for fish to leave the area of danger, the turbidity of the sulphidic water may still facilitate escape at these shallow depths. We note that another possible explanation is that fish did not perceive the threat of the bimodal stimulus as strong enough to show maximal dive responses. While we cannot fully exclude this explanation with the data at hand, the uniformity in dive times between our lab and field experiments as well as those observed after natural bird attacks (personal observation) make longer and deeper dives appear highly unlikely. Nonetheless, we recommend examining the roles that cue quantity and quality as well as other modalities, especially hydrodynamic movement cues, play in the detection and evasion of aerial predation.

The observed pattern of unselective dive initiation with subsequent fine-tuning of diving parameters appears analogous to multistage antipredator responses (e.g., Hemmi and Pfeil 2010). By performing subsequent behaviors, animals can reduce risk, update or gather new information and thus optimize their performance during each step. We propose a similar mechanism here, in which fish maintain a high cue sensitivity initially (low false-negative rate), but then reduce the costs of responses when not at greatest danger (reducing costs of false positives). This mechanism would enable sulphur mollies to quickly return to the oxygenated surface, and consequently keep costs of energy expenditure and missed opportunities to a minimum. More generally, this may be adaptive for any species that faces the conflicting demands of the air-water interface being source of both, potential predation and resource acquisition (e.g., aquatic insects depending on atmospheric oxygen; Rodríguez-Prieto et al. 2006). For fish’s fast-start dives, it remains an open question whether individuals actively increase or decrease their performance during stages of the fast-start that are under cognitive behavioral control, and experiments that expose fish to different cost-benefit ratios (in the case of the sulphur molly, e.g., through exposure to varying levels of hypoxia; see Lukas, Auer, et al. 2021) may help to investigate this further. As such, our study may serve as a starting point for future investigations in this system, which provides an ideal ground to explore how animals optimize their abilities to reduce costs of nonadaptive responses (i.e., false positives and false negatives), be it through adjusting individual response performance or collective information processing (Sosna et al. 2019).

## SUPPLEMENTARY MATERIAL

Supplementary data are available at *Behavioral Ecology* online.

Video 1 Sulphur mollies (*Poecilia sulphuraria*) form large shoals and typically react to above-surface disturbances with rapid, seconds-lasting diving into the oxygen-free water column. Underwater recording (GoPro Hero 6) taken at Baños del Azufre, where fish were exposed to an artificial visual predator stimulus.

Video 2 The nature of a disturbance – whether it is an approaching bird predator, an overflying one without attack intention or a non-piscivore– is hard to identify on the basis of visual cues alone. Underwater recording (GoPro Hero 6) taken at Baños del Azufre, where a great-tailed grackle (*Quiscalus mexicanus*) flies over a shoal of sulphur mollies (*Poecilia sulphuraria*).

arab043_suppl_Supplementary_Material

arab043_suppl_Supplementary_Video_1

arab043_suppl_Supplementary_Video_2

## FUNDING

This work was supported by the Deutsche Forschungsgemeinschaft (DFG; BI 1828/3-1 (DB), RO 4766/2-1 (PR), EXC 2002/1 “Science of Intelligence” project 390523135 [J.K., P.R., and D.B.]) and the Open Access Publication Fund of the Humboldt-Universitä zu Berlin. J.L. was partially supported by the Berlin Funding for Graduates (Elsa-Neumann-Scholarship des Landes Berlin).

We thank M. Sroka, C. Twomey, C. Doran, M. Habedank, D. Lewis, J. Petrasch, J. Bak-Coleman, A. Jordan, G. Mazué, and G. Rieuceau for help with fieldwork. We are grateful to the director and staff at the *CIIEA Centro de Investigación e Innovación para la Enseñanza y el Aprendizaje* field station in Teapa (Mexico) for hosting our multiple research stays. J. Choi, J. Piotrowski, and C. Schutz assisted with video tracking. In addition, we thank K. Laskowski for helpful advice on data analysis as well as T. Mehner, M. Alirangues Nuñez, F. Dhellemmes, F. Kupprat, D. Neubauer, and G. Scholtysik for discussion of an earlier manuscript draft. We thank the editor, and two reviewers for their constructive suggestions that improved the manuscript. All procedures adhered to the “Guidelines for the treatment of animals in behavioral research and teaching” (Animal Behaviour 2020) and were approved by the Mexican government (DGOPA.09004.041111.3088, PRMN/DGOPA-009/2015, and PRMN/DGOPA-012/2017 issued by SAGARPA-CONAPESCA-DGOPA).

## CONFLICT OF INTEREST

The authors declare that no conflict of interest exists.

## AUTHOR CONTRIBUTIONS

J.L., P.R., and D.B. performed the laboratory experiment. Field data on bird activity and fish’s dive response was collected by J.L., J.K., and D.B. with assistance from all other authors. P.R., P.K., and H.K. contributed tools for acoustic predator stimulation and video analysis. J.L. performed the data and statistical analysis and wrote the manuscript with input from all authors. All authors gave final approval for publication.

## Data availability

Analyses reported in this article can be reproduced using the data and R-code provided by Lukas and colleagues (2021).
