# Supplementary material for: Acoustic and visual stimuli combined promote stronger responses to aerial predation in fish
Source: Behav Ecol. 2021 Jul 19;32(6):1094–102. doi: 10.1093/beheco/arab043 (PMC8691536; doi:10.1093/beheco/arab043)
Supplement: arab043_suppl_Supplementary_Material [file arab043_suppl_supplementary_material.docx]

# Supplement

## 1. Natural observations of bird cues

### 1.1 Predator hunting strategies

*Plunge-diving kingfishers:*

Prior to an attack, kingfishers often perch near or above the water, where they can stay hidden against background vegetation. When they launch an attack, they fly in a direct line for their prey. As they close the distance to the water, kingfishers fold back their wings thus exposing their white underbelly. Like a projectile, kingfishers plunge-dive into the water after their prey, fully submerging their body and thus greatly disturbing the water upon impact.

*Wading shorebirds:*

Herons, egrets, bitterns, stilts, grackles, jays, sandpipers and plovers are often found wading along shore or in shallow water, where they search for prey by probing the ground with their beaks and feet. Early detection of these predators is likely dependent on their size (e.g., small plovers and sandpipers remain undetectable for longer) and movement. Snowy egrets are often seen running, during which they flap their wings, causing lots of movement and water waves. In stark contrast, green herons remain almost motionless before they ambush their prey.

*Shallow-water divers:*

Neotropical cormorants forage by pursuit-diving. Often observed hunting in pairs, they herd the prey fish together, causing lots of commotion in the water during diving. During periods of recovery, cormorants either float at the surface or leave the water for extended periods as they wing-dry ashore.

*Mid-flight capture:*

Great kiskadees are skilled insect catchers, but also feed on fish. Despite their flashy yellow bellies, these birds manage to stay hidden by selecting perches higher up in the bushes or trees. When launching an attack, they sally out of hiding and pluck fish from the water’s surface. As merely their beak enters the water, these birds cause no to very little disturbance to the water during their low flight.

### 1.2. Natural observations of bird cues: occurrence of attacks and attack-unrelated overflights

SI Table 1: Overview of candidate models to determine the frequency of bird disturbance events. Results of model selection based on GLMs. Variables tested include an effect of disturbance type, as well as sampling time. Degrees of freedom (df), Akaike’s Information Criterion for finite sample sizes (AICc), Delta AIC (ΔAICc) and model weights (w_i_) are listed. The top ranked model (lowest AICc) and models with equal support (ΔAICc < 2) are in bold.

| Candidate models | *AICc* | *ΔAICc* | *df* | *w_i_* |
| --- | --- | --- | --- | --- |
| M1: (Intercept) | 210.3 | 16 | 2 | <0.001 |
| M2: (Intercept) + type | 201.9 | 7.7 | 3 | 0.02 |
| M3: (Intercept) + sampling | 206.0 | 11.7 | 4 | 0.002 |
| M4: (Intercept) + type + sampling | 198.4 | 4.1 | 5 | 0.11 |
| **M5: (Intercept) + type * sampling** | **194.2** | **0** | **7** | **0.87** |

SI Table 2: Frequency of attacks and overflights at different sampling times. Shown are the results of a generalized linear model (M5, SI Table 1). Intercept taken at disturbance type [overflight] and sampling [morning]. Bold entries indicate significance with *P* < 0.05.


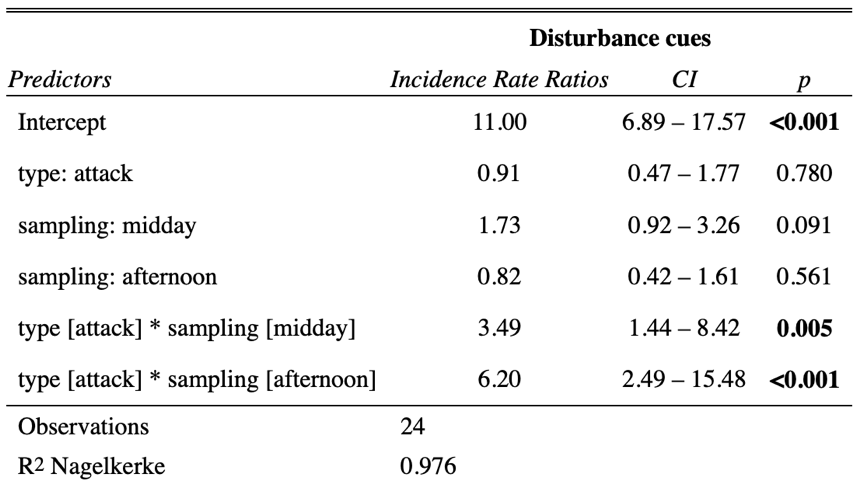


## 2. Field observations on the diving behavior of *P. sulphuraria*

### 2.1 Response to predator stimuli

SI Table 3: Dive times in response to an artificial visual predator stimulus. Shown are the results of a linear mixed model (see main manuscript). Intercept taken at exposure = 1.


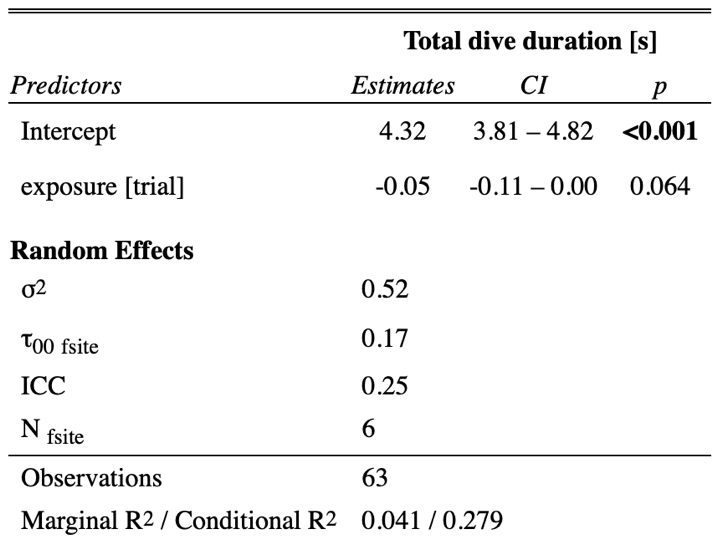


## 3. Laboratory study

### 3.1 Anti-predator diving: individual diving parameters

SI Table 4: Overview of candidate models to determine the effect of predator stimuli on anti-predator diving behavior of *P. sulphuraria*. Results of model selection based on (G)LMMs. Variables tested include an effect of predator stimulus, as well as stimulus exposure (trial). Degrees of freedom (df), Akaike’s Information Criterion for finite sample sizes (AICc), Delta AIC (ΔAICc) and model weights (w_i_) are listed. The top ranked model (lowest AICc) and models with equal support (ΔAICc < 2) are in bold.

| Candidate models | **Responsiveness  [Prop. of divers]** | | | | **Fast-start depth**  **[cm]** | | | | **Fast-start duration**  **[s]** | | | |
| --- | --- | --- | --- | --- | --- | --- | --- | --- | --- | --- | --- | --- |
|  | *AICc* | *ΔAICc* | *df* | *w_i_* | *AICc* | *ΔAICc* | *df* | *w_i_* | *AICc* | *ΔAICc* | *df* | *w_i_* |
| M1: (Intercept) | 196.5 | 20 | 3 | <0.001 | 657.8 | 95 | 3 | <0.001 | -50.6 | 111.1 | 3 | <0.001 |
| **M2: (Intercept) + stimulus** | **176.5** | **0** | **5** | **0.65** | **562.8** | **0** | **5** | **0.66** | **-161.7** | **0** | **5** | **0.57** |
| M3: (Intercept) + trial | 198.6 | 22.1 | 4 | <0.001 | 659.3 | 96.5 | 4 | <0.001 | -48.7 | 113 | 4 | <0.001 |
| **M4: (Intercept) + stimulus + trial** | **178.6** | **2.2** | **6** | **0.22** | **564.5** | **1.7** | **6** | **0.29** | **-160.2** | **1.5** | **6** | **0.27** |
| M5: (Intercept) + stimulus * trial | 179.8 | 3.3 | 8 | 0.13 | 567.9 | 5.1 | 8 | 0.05 | -159.1 | 2.6 | 8 | 0.16 |

| Candidate models | **Fast-stat maximum speed [cm/s]** | | | | **Total dive duration**  **[log(s)]** | | | |
| --- | --- | --- | --- | --- | --- | --- | --- | --- |
|  | *AICc* | *ΔAICc* | *df* | *w_i_* | *AICc* | *ΔAICc* | *df* | *w_i_* |
| M1: (Intercept) | 872.4 | 74.8 | 3 | <0.001 | 124.6 | 25.8 | 3 | <0.001 |
| M2: (Intercept) + stimulus | 800.4 | 2.8 | 5 | 0.18 | 106.9 | 8.2 | 5 | 0.01 |
| M3: (Intercept) + trial | 869.6 | 72 | 4 | <0.001 | 119.7 | 21 | 4 | <0.001 |
| **M4: (Intercept) + stimulus + trial** | **797.6** | **0** | **6** | **0.74** | **98.8** | **0** | **6** | **0.86** |
| M5: (Intercept) + stimulus * trial | 802 | 4.5 | 8 | 0.08 | 102.6 | 3.8 | 8 | 0.13 |

SI Table 5: Effect of predator stimuli on anti-predator diving behavior of sulphur mollies. Shown are the results of (general) linear mixed models (M4, SI Table 4) Intercepts taken at predator stimulus [visual] and exposure [1]. Bold entries indicate significance with *P* < 0.05.


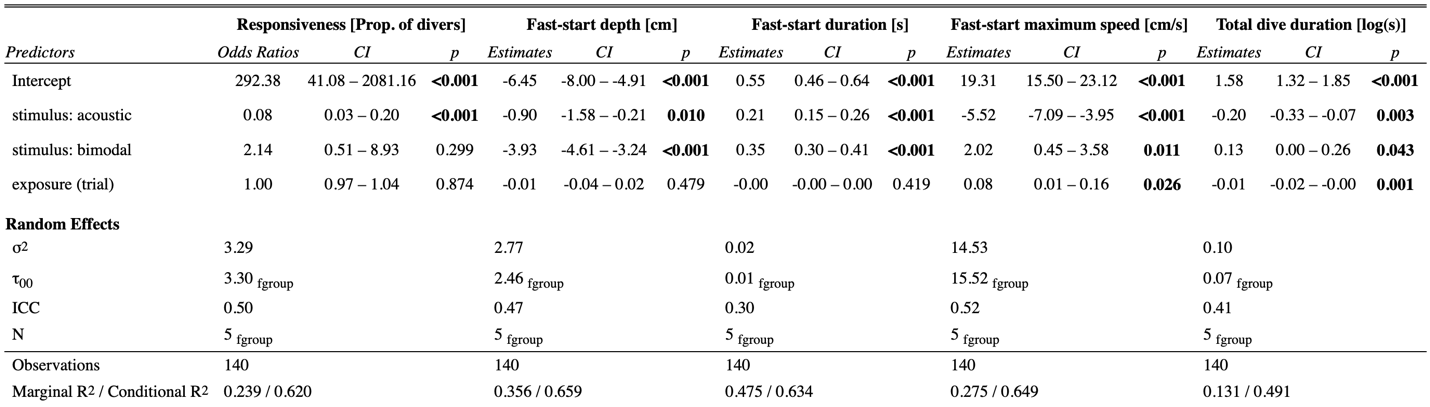


SI Table 6: Pairwise comparisons between predator stimuli. Shown are post-hoc tests on estimated marginal means with ‘mvt’ adjustment (see SI Tables 4,5 for model specifications). Bold entries indicate significance with *P* < 0.05.

| **Comparison** |  | Mean difference | Significance(mvt) |
| --- | --- | --- | --- |
| *Responsiveness [Prop. of divers]* |  |  |  |
| unimodal visual *vs.* bimodal |  | 2.14 | 0.299 |
| unimodal acoustic *vs.* bimodal |  | 27.11 | **< 0.001** |
| *Fast-start depth [cm]* |  |  |  |
| unimodal visual *vs.* bimodal |  | -3.93 | **< 0.001** |
| unimodal acoustic *vs.* bimodal |  | -3.03 | **< 0.001** |
| *Fast-start duration [s]* | | | |
| unimodal visual *vs.* bimodal |  | 0.35 | **< 0.001** |
| unimodal acoustic *vs.* bimodal |  | 0.15 | **0.002** |
| *Fast-start maximum speed [cm/s]* | | | |
| unimodal visual *vs.* bimodal |  | 2.02 | **0.013** |
| unimodal acoustic *vs.* bimodal |  | 7.54 | **< 0.001** |
| *Total dive duration [s]* | | | |
| unimodal visual *vs.* bimodal |  | 1.14 | **0.045** |
| unimodal acoustic *vs.* bimodal |  | 1.40 | **< 0.001** |
|  |  |  |  |

### 3.2 Anti-predator diving: group performance

We explored two group performance scores: *cohesion* as a measure of how unified fish dove in the y-plane, and *polarization* to quantify how aligned fish were in the x-y-plane. However, as the experimental set-up only allowed for 2D-tracking, these preliminary results need to be confirmed with 3D-analysis.

Both variables were calculated for the period in which all 12 individuals were diving, starting from the last fish of the group initiating its dive until the first fish resurfaced. Cohesion was quantified via the coefficient of variance of y-positions of all 12 individuals, with values below 1 indicating low variance.

We computed polarization as the sum of the unit velocity vectors of all 12 individuals, resulting in a mean of sums ranging from 0–1, with the value 1 signifying all fish moving (e.g. diving) in the same direction, while values close to 0 indicate movement in random directions that cancel each other out. However, by definition the movement of a collective diving response cannot be entirely random and values close to zero (i.e. < 0.25) are extremely unlikely. Model construction of cohesion and polarization followed the methodology outlined in the main manuscript (log-normal error and REML estimation).

Overall, groups showed little variance in terms of depth distribution among group members (cohesion: > 0.45) and a tendency for parallel alignment during diving (polarization: > 0.5). Bimodal predator stimuli resulted in higher levels of cohesion (i.e. lower variance) and polarization relative to single-stimulus treatment (cohesion: *F*_2,135_ = 8.0, *P* < 0.001; polarization: *F*_2,135_ = 11.3, *P* < 0.001; SI Fig. 1A/B). We found no evidence for stimulus-specific or overall habituation to the repeated exposure on either parameter (stimulus × exposure interaction and covariate exposure, based on AICc scores; SI Table 7).

**
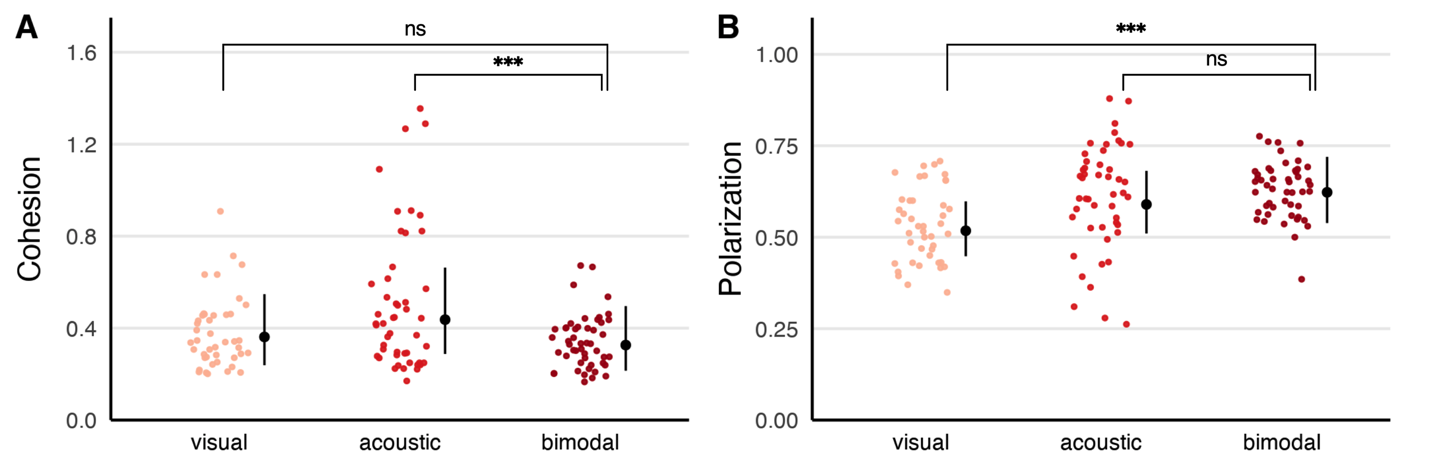
**

SI Figure 1: Effect of predator stimuli on fish’s diving behavior (group-level). A-B: Stimulus type affected group’s cohesion and polarization during diving (*P* < 0.05; for details see SI text and SI Tables 7,8). Shown are group-pooled means (*n* = 5 groups of 12 fish) with model-estimated marginal means ± 95% CI. Asterisks indicate results from post hoc pairwise comparisons (SI Table 9).

SI Table 7: Overview of candidate models to determine the effect of predator stimuli on anti-predator diving behavior of *P. sulphuraria*. Results of model selection based on LMMs. Variables tested include an effect of predator stimulus, as well as stimulus exposure (trial). Degrees of freedom (df), Akaike’s Information Criterion for finite sample sizes (AICc), Delta AIC (ΔAICc) and model weights (w_i_) are listed. The top ranked model (lowest AICc) and models with equal support (ΔAICc < 2) are in bold.

| Candidate models | **Cohesion [log]** | | | | **Polarization** | | | |
| --- | --- | --- | --- | --- | --- | --- | --- | --- |
|  | *AICc* | *ΔAICc* | *df* | *w_i_* | *AICc* | *ΔAICc* | *df* | *w_i_* |
| M1: (Intercept) | 148.2 | 11.1 | 3 | 0.003 | -31.1 | 16.3 | 3 | <0.001 |
| **M2: (Intercept) + stimulus** | **137.1** | **0** | **5** | **0.64** | **-47.3** | **0.2** | **5** | **0.45** |
| M3: (Intercept) + trial | 149.6 | 12.5 | 4 | 0.001 | -31 | 16.5 | 4 | <0.001 |
| **M4: (Intercept) + stimulus + trial** | **139** | **1.8** | **6** | **0.26** | **-47.4** | **0** | **6** | **0.49** |
| M5: (Intercept) + stimulus * trial | 140.9 | 3.8 | 8 | 0.1 | -43 | 4.4 | 8 | 0.05 |

SI Table 8: The effect of predator stimuli on anti-predator diving behavior of sulphur mollies from 5 groups. Shown are the results of linear mixed models (M4; SI Table 7). Intercepts taken at predator stimulus [visual] and exposure [1]. Bold entries indicate significance with *P* < 0.05.


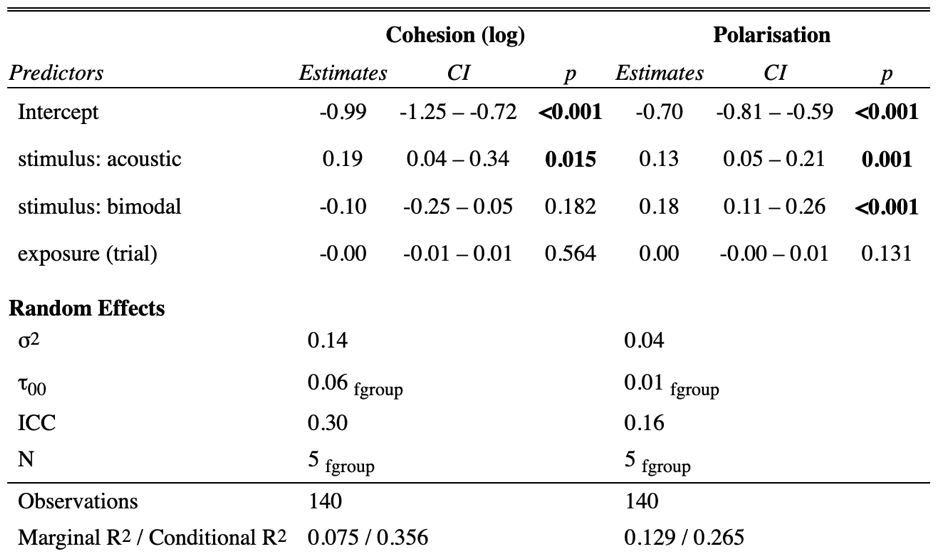


SI Table 9: Pairwise comparisons between predator stimuli. Shown are post-hoc tests on estimated marginal means with ‘mvt’ adjustment (see SI Tables 7,8 for model specifications). Bold entries indicate significance with *P* < 0.05.

| **Comparison** |  | Mean difference | Significance(mvt) |
| --- | --- | --- | --- |
| *Cohesion(log)* |  |  |  |
| unimodal visual *vs.* bimodal |  | -0.10 | 0.185 |
| unimodal acoustic *vs.* bimodal |  | -0.29 | **< .001** |
| *Polarization* |  |  |  |
| unimodal visual *vs.* bimodal |  | 0.18 | **<.001** |
| unimodal acoustic *vs.* bimodal |  | 0.05 | 0.164 |
|  |  |  |  |

### 3.3 Body size estimation of test fish

For the experiment, test fish were visually matched for size. To verify that groups did not significantly differ in size, body sizes were estimated from video images taken during the experiment (SI Fig. 2). For each group 10 images were analyzed using the image processing program ImageJ. To minimize a systematic error introduced through fish’s distribution in the z-axis (i.e. distance to camera), measurements were only taken for individuals that were very close to the tank’s front glass and not occluded by another fish. This resulted in 4–9 (opposed to 12) individuals being measured for standard length to the nearest millimeter. Pixel values were then converted back to real-world distances using known tank dimensions. On average, test fish were estimated to be 15 mm, ranging from 13 to 17 mm.


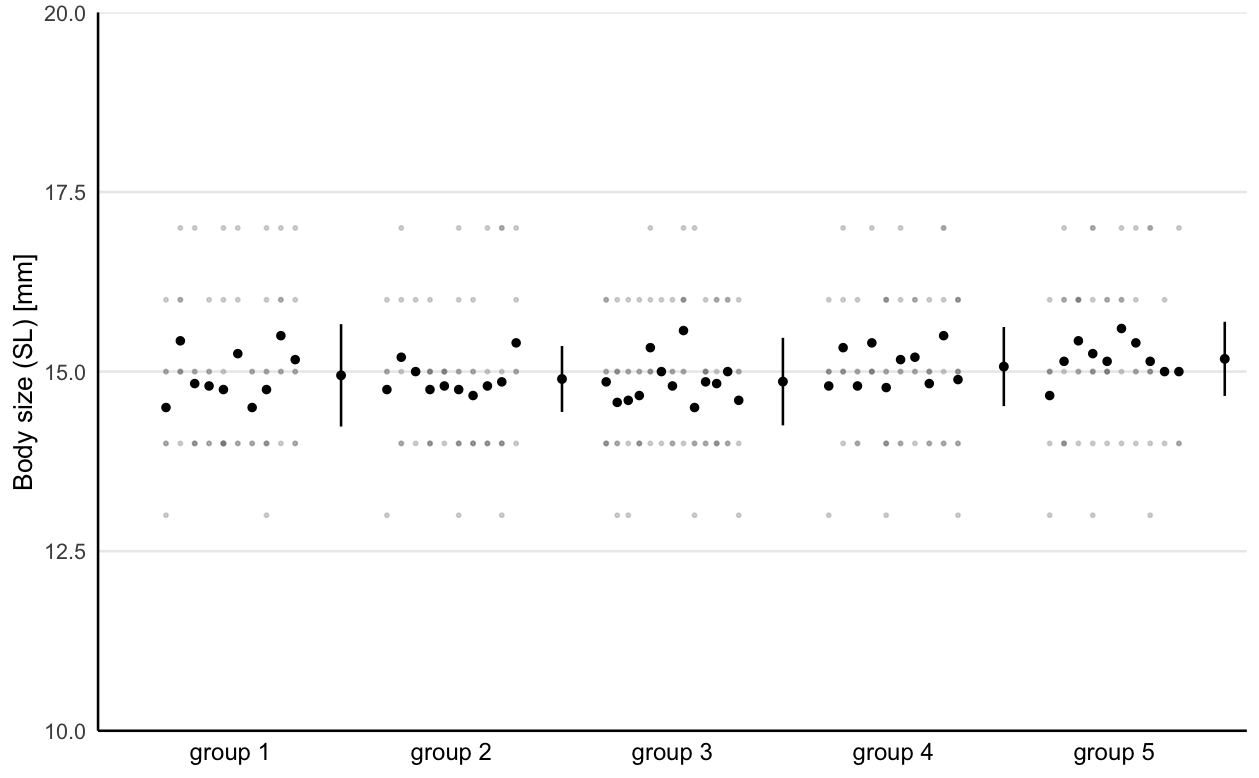


SI Figure 2: Estimated body sizes of test fish. Shown are raw estimates (grey) and image-pooled means (black, n = 10 images of 4–9 measurements) with model-estimated marginal means ± 95% CI.
